# Succinylation of Apolipoprotein E Drives Alzheimer’s Disease

**DOI:** 10.1002/alz70861_108681

**Published:** 2025-12-23

**Authors:** Nicholas A Devanney

**Affiliations:** ^1^ Buck Institute for Research on Aging, Novato, CA USA

## Abstract

**Background:**

The ε4 allele of apolipoprotein E (*APOE*) is the strongest genetic risk factor for late‐onset Alzheimer’s disease (LOAD). Impaired mitochondrial function disrupts metabolic homeostasis in the brains of ε4 carriers decades prior to disease onset. Microglia and astrocytes expressing ε4 display a ‘broken’ TCA cycle, including elevated levels of the metabolite succinate. However, it is unknown whether this accumulation of succinate in ε4 may lead to increased succinylation, a recently discovered post‐translational modification which dramatically alters protein function by introducing four carbons and a ‐2 charge.

**Method:**

Succinylated peptides were enriched from hippocampi of 12‐month old male 5xFAD mice and wild‐type controls using pan‐succinyllysine magnetic beads (PTMScan) and detected by LC‐MS/MS on the Orbitrap Eclipse Tribid mass spectrometer platform (Thermo Fisher Scientific) operating in data‐independent acquisition mode. Data was processed using directDIA (Spectronaut).

**Result:**

588 succinylated peptides were quantified in the hippocampus of 5xFAD and wild‐type mice. Of these, 3 succinylation sites were detected for apoE including lysine K252, located within the lipid‐binding domain in a region known to be critical for apoE self‐aggregation and amyloid plaque seeding. Succinylation at K252 increased >8‐fold in 5xFAD hippocampi relative to wild‐type controls (*p* =0.02).

**Conclusion:**

Studies on the posttranslational modification of apoE have so far focused primarily on its glycosylation. Little is known about other PTMs that may confer disease risk in ε4 carriers. Our discovery that succinylation of apoE is increased in the 5xFAD hippocampus suggests that this modification occurs in response to disease pathology and opens the door to a new mechanism by which apoE function is regulated across the disease trajectory. We hypothesize that succinylation of apoE impacts functions critical for neuroprotection against AD in an allelic‐dependent manner, and are currently investigating whether modulation of succinylation represents a novel therapeutic approach for AD.

